# Proteomic Analysis of Liver Injury Induced by Deoxynivalenol in Piglets

**DOI:** 10.3390/biology14121721

**Published:** 2025-12-01

**Authors:** Xiaoshu Xue, Ping Wu, Shuhao Fan, Zongjun Yin, Xiaodong Zhang

**Affiliations:** College of Animal Science and Technology, Anhui Agricultural University, Hefei 230036, China; xuexiaoshu0812@126.com (X.X.); shuhaowudi@163.com (S.F.); yinzongjun@ahau.edu.cn (Z.Y.)

**Keywords:** deoxynivalenol, pig, liver, mycotoxins, proteomics

## Abstract

Deoxynivalenol (DON, commonly known as vomitoxin) is one of the most prevalent mycotoxins contaminating feed in China, posing a serious threat to the health of piglets. Beyond intestinal damage, the liver is a key target organ for the systemic toxicity of DON, but its hepatotoxic molecular mechanisms, particularly the changes at the proteome level, remain unclear. To investigate the protein regulatory network of DON-induced liver injury in piglets, this study systematically analyzed differential expression in the liver proteome using quantitative proteomic techniques. Proteomic analysis identified 5851 proteins in total, among which 88 were differentially expressed proteins (DEPs), including 39 upregulated and 49 downregulated proteins. Bioinformatics analysis revealed that these DEPs were significantly enriched in pathways such as DNA damage repair, RNA metabolism, ribosome biogenesis, and cysteine metabolism. Suppressed expression of key proteins like Replication Factor C Subunit 4 (RFC4) and Exosome Component 9 (EXOSC9) indicated that DON exposure severely disrupted the maintenance of genomic stability and RNA processing capacity in hepatocytes. Conversely, the activation of Nucleic Acid Binding Protein 1 (NABP1) might represent a compensatory DNA protection response. Furthermore, the upregulation of Lactate Dehydrogenase B (LDHB) suggested that DON might influence epigenetic modifications by regulating lactate metabolism. This study reveals, for the first time from a proteomic perspective, a novel mechanism by which DON induces hepatotoxicity in piglets by disrupting DNA repair and RNA metabolic homeostasis, providing an important theoretical basis and data support for elucidating the toxicological effects of DON and improving feed biosafety control strategies.

## Simple Summary

Piglets are highly sensitive to the residual DON in feed, but the mechanism of how DON damages the liver at the protein level remains unknown. We utilized proteomics to analyze the effects of DON exposure for 28 days on the livers of piglets and identified some potential toxicological targets, with the aim of providing therapeutic strategies to improve liver health in piglets.

## 1. Introduction

Deoxynivalenol (DON), commonly known as vomitoxin, is a type of trichothecene mycotoxin produced by fungi of the *Fusarium* genus (e.g., Fusarium *graminearum*) [1]. This toxin is named for its strong emetic effect in pigs and is one of the most commonly contaminating mycotoxins in grains [2,3]. If grains are not harvested promptly during the late growth stage or are stored under conditions of high temperature and humidity, Fusariumfungi can proliferate and produce secondary metabolites such as DON [4,5]. As the primary raw material for feed, the safety of grains directly affects animal health and agricultural production efficiency. The humid climates of China’s Yangtze and Yellow River basins and southern regions provide suitable conditions for the growth of *Fusarium* [6]. Surveys indicate that the detection rate of DON contamination in Chinese feed and raw materials exceeds 90%, with average (0.9–2.3 mg/kg) contamination levels significantly higher than the European Union’s maximum limit standards (0.9 mg/kg, Directive 2006/576/EC), posing an ongoing challenge to China’s aquaculture and feed safety sectors [7,8]. Besides its widespread contamination, DON also possesses highly stable chemical properties. The 12,13-epoxy group in its molecular structure is the core of its toxicity and confers strong thermal and acid-base stability [9,10]. Studies show that DON can retain over 50% of its activity even after treatment at 210 °C and its structure remains stable when heated at 100 °C under pH = 4 conditions for 60 min [11]. Therefore, conventional food processing methods such as baking, steaming, and extrusion are ineffective in significantly degrading DON. Furthermore, DON exists in several acetylated derivatives, such as 3-acetyl-DON (3-ADON) and 15-acetyl-DON (15-ADON) [10]. These derivatives can be metabolized into DON by gut microbiota or the liver, exhibiting toxicity close to or slightly lower than DON while possessing excellent chemical stability [12]. Although some methods for effective DON degradation (e.g., microbial enzymatic hydrolysis, montmorillonite adsorption, ozone treatment) have been developed and have achieved certain application results in the food field, due to high processing costs, unstable efficiency, and potential impacts on feed nutritional value, these technologies have not yet been widely adopted for large-scale detoxification of livestock feed [11,13].

Among various livestock, piglets are the most sensitive to the toxicity of DON [3]. Its emetic mechanism is closely related to the regulation of neurotransmitter-like substances and vagus nerve stimulation [14]. Low-dose DON exposure (>1 mg/kg) can induce vomiting, diarrhea, and growth retardation in piglets, while high doses (>10 mg/kg) may lead to immunosuppression and even death [15]. Current research generally suggests that DON’s toxicity primarily occurs through binding to the peptidyl transferase center, inhibiting protein synthesis, and activating Mitogen-Activated Protein Kinase (MAPK) and Nuclear Factor Kappa-B (NF-κB) inflammatory pathways, thereby causing damage to the intestinal mechanical and immune barriers [16]. However, although the intestine is the main barrier for the absorption of exogenous toxins, absorbed DON can still enter the liver via the portal vein system. As the central organ for substance metabolism and xenobiotic detoxification, the liver becomes a key target organ for the in vivo distribution and toxic effects of DON [17,18]. Long-term, low-dose DON exposure can lead to hepatocellular vacuolar degeneration, fat deposition, perivenular fibrosis, inflammatory cell infiltration, and alter Cytochrome P450 (CYP450) enzyme activity, disrupting endogenous metabolic homeostasis [19].

At the molecular mechanism level, several transcriptomic studies in recent years have revealed gene expression changes associated with DON-induced liver injury, involving pathways related to oxidative stress, apoptosis, lipid metabolism, and immune inflammation [20,21]. However, multi-omics consistency studies indicate that the correlation between mRNA and protein levels in biological systems is only about 46%, suggesting that transcriptomic data alone cannot fully reflect the actual protein dynamics and functional manifestations of toxic effects [22]. Factors such as post-transcriptional regulation, protein translational modifications, and degradation rates can all lead to discrepancies between mRNA and protein expression. Therefore, conducting toxicological mechanism research directly at the protein level is of great significance.

Based on this, we hypothesized that DON exposure induces hepatotoxicity in piglets by disrupting DNA damage repair and RNA metabolism pathways. We employed Tandem Mass Tag (TMT)-based quantitative proteomics technology, combined with Parallel Reaction Monitoring (PRM) targeted validation, to analyze the global proteome expression profile of liver tissue from DON-exposed piglets. The aim was to systematically screen for differentially expressed proteins (DEPs) and reveal key toxicological pathways and molecular mechanisms through bioinformatics analysis. The results are expected to deepen the understanding of DON hepatotoxicity at the protein level and provide a theoretical basis for the subsequent development of detoxification strategies and feed safety early warning systems.

## 2. Materials and Methods

### 2.1. Experimental Design

Following the previously described method, two types of diets were prepared: a control diet and a diet contaminated with DON, ensuring that the DON level (basal diet supplemented with 4 mg/kg DON, ≥98% purity, Sigma-Aldrich, Darmstadt, Germany) in the contaminated feed exceeded the maximum limit set by the national standard (GB13078-2017 [23]). A total of twelve 35-day-old weaned piglets were randomly allocated into two groups *(n* = 6 per group). Apart from being fed the different diets, all piglets were housed under identical standard conditions with ad libitum access to water and feed. The experimental diets were formulated in accordance with the guidelines of the Nutrient Requirements of Swine (NRC, 2012 [24]) and Nutrient requirements of swine (GB/T39235-2020 [25]).

The feeding trial lasted for 28 days. Blood samples were obtained from the jugular vein prior to morning feeding at 28 dpi. Samples were centrifuged at 3000 rpm for 15 min to separate serum and plasma, which were stored in liquid nitrogen. Subsequently, the piglets were euthanized via intravenous injection with a lethal dose of sodium pentobarbital (Sinopharm, Beijing, China). Immediately after euthanasia, necropsy was performed, liver samples were harvested from the left lobes of pigs within 30 min. A portion of the liver sample was fixed in 4% paraformaldehyde (PFA) for subsequent histological analysis, while another portion was rapidly frozen in liquid nitrogen and stored at −80 °C for further molecular analyses.

### 2.2. Protein Extraction

The sample was transferred to a grinding tube. A volume of 400 μL of 8 M urea solution and 4 μL of phenylmethanesulfonyl fluoride (PMSF) reagent were added to the sample using an accurate pipetting technique. The mixture was homogenized using a mechanical grinder with the following cycle: 1 min grinding followed by 1 min pause, repeated for a total of 12 cycles until a homogeneous paste was obtained. The homogenate was centrifuged at 12,000 rpm and 4 °C for 10 min. The resulting supernatant was carefully transferred to a pre-cooled 1.5 mL microcentrifuge (EP) tube. Subsequently, 200 μL of 8 M urea solution and 2 μL of PMSF reagent were added to the residual pellet in the grinding tube. The mixture was subjected to additional homogenization using the same grinding protocol (1 min grinding/1 min pause) for 6 cycles. Following homogenization, the sample was centrifuged again under identical conditions (12,000 rpm, 4 °C, 10 min). The supernatant was collected and combined with the previously obtained supernatant in the 1.5 mL EP tube. The pooled supernatant was mixed thoroughly by gentle inversion or brief vortexing and stored on ice for subsequent analysis. Protein concentration in the final extract was determined using the bicinchoninic acid (BCA) protein assay according to the manufacturer’s instructions.

### 2.3. Protein Sample Preparation, Digestion, and Peptide Clean-Up

For each sample, 100 μg of protein solution was aliquoted into a 1.5 mL tube and diluted to 100 μL with 50 mM ammonium bicarbonate (NH_4_HCO_3_). The proteins were reduced with 10 mM dithiothreitol (DTT, final concentration) at 56 °C for 1 h, followed by alkylation with 20 mM iodoacetamide (IAM, final concentration) in the dark at room temperature for 40 min. Excess IAM was quenched by adding DTT to a final concentration of 10 mM.

Subsequently, the proteins were purified and digested using a single-pot solid-phase-enhanced sample preparation (SP3) method. Briefly, a 1:1 mixture of hydrophilic and hydrophobic magnetic beads was washed three times with ultrapure water. For each 100 μg of protein sample, 10 μL of the bead slurry was added along with 110 μL of absolute ethanol to facilitate binding. The mixture was incubated with continuous mixing at room temperature for 15 min. After binding, the supernatant was removed using a magnetic stand, saved, and labeled as the flow-through. The beads were then washed three times with 500 μL of 80% ethanol. After complete removal of the wash solution, the beads were briefly dried (until the bead surface appeared rough without liquid reflection, while avoiding over-drying and cracking). The beads were resuspended in 300 μL of 50 mM NH_4_HCO_3_. Trypsin was added at an enzyme-to-protein ratio of 1:50 (*w*/*w*), and digestion was carried out at 37 °C with shaking at 1000 rpm overnight (14–18 h).

Following digestion, the peptide-containing supernatant was collected using a magnetic stand. The peptides were concentrated by lyophilization. The dried peptides were further desalted using C18 StageTips and vacuum-dried at 45 °C prior to Liquid Chromatography-Tandem Mass Spectrometry (LC-MS/MS) analysis.

### 2.4. Liquid Chromatography-Mass Spectrometry Analysis

Chromatographic separation was performed using a nanoflow liquid chromatography system. Peptides were first loaded onto a pre-column (PEPMAP NEO C18, 300 μm × 5 mm) and then separated on an analytical column (5.5 cm High Throughput uPACTM Neo HPLC Column). The mobile phases consisted of (A) 0.1% formic acid in water and (B) 0.1% formic acid in 80% acetonitrile. The separation was carried out at a constant flow rate of 2.5 μL/min with a total run time of 6.9 min per sample. A linear gradient was applied as follows: 4% to 20% B (0–4 min), 20% to 35% B (4–5.8 min), 35% to 99% B (5.8–6.2 min), held at 99% B for 0.7 min (6.2–6.9 min).

The eluted peptides were analyzed by a tandem mass spectrometer operating in data-dependent acquisition (DDA) mode. The key mass spectrometry parameters were set as follows:

Full MS (MS1): The resolution was set to 240,000. The automatic gain control (AGC) target was 500% with a maximum injection time of 5 ms. The scan range was from 380 to 980 *m*/*z*. MS2 resolution was set to 30,000 at *m*/*z* 200 using HCD fragmentation (manufacturer’s default). The AGC target was 500% with a maximum injection time of 3 ms. The scan range was from 380 to 980 *m*/*z*, and a normalized collision energy of 25% was applied.

### 2.5. Database Search

The raw mass spectrometry data were analyzed using Proteome Discoverer 2.4 (Thermo Fisher Scientific, Waltham, MA, USA) with the SEQUEST HT search engine. The TMT-labeled spectra were acquired under data-dependent acquisition (DDA) mode.

### 2.6. Bioinformatics Analysis

The protein intensity values were normalized using the median normalization method to minimize technical variances arising from sample loading and instrument operation. Missing values were imputed using a half-minimum value approach. To identify differentially expressed proteins (DEPs), the fold change (FC) for each protein was calculated as the ratio of the mean intensity between the two compared sample groups. The quality parameters, Venn diagram, volcano plots, and hierarchical clustering were obtained by one-way analysis of variance, *t*-test, and R-software (Rstudio 4.5). Furthermore, gene set enrichment analysis (GSEA) was used to cluster significant gene sets associated with given annotation terms using the criteria was FRD > 0.25. Gene ontology (GO) analysis was performed using the DAVID database. Benajmini correction of *p* < 0.05 was used to determined significance. The pathways enrichment analysis was based on the Reactome database, and protein–protein interaction (PPI) networks were performed by Cytoscape (3.10.4) using the ClueGO plugin.

### 2.7. Western Blot (WB) Analysis

Details of WB method was described in our previous paper [26]. Antibodies of APOE and Tubulin were purchased from Huabio (China, Hangzhou) with Catalog# HA721867 and HA721913. Antibodies of SGTB, NABP1 and RFC4 were purchased from Abcam (UK, Cambridge) with Catalog# ab202419, ab236402 and ab182145.

### 2.8. Elisa Analysis

ALT and AST were determined using a commercially available assay kit (Meimian, Yancheng, China) via the ELISA method with Catalog# MM-36264O2 and MM-36265O2. The sensitivity of the porcine ALT and AST ELISA kit is 0.16 ng/mL, with a detection range of 0.313 to 20 ng/mL. The coefficient of variation (CV) within the plate is less than 10%, and between plates is less than 15%. Data were analyzed using Student’s *t*-test with *p* < 0.05 considered significant.

## 3. Results

### 3.1. DON Treatment Causes Porcine Liver Injury

**Figure 1:**
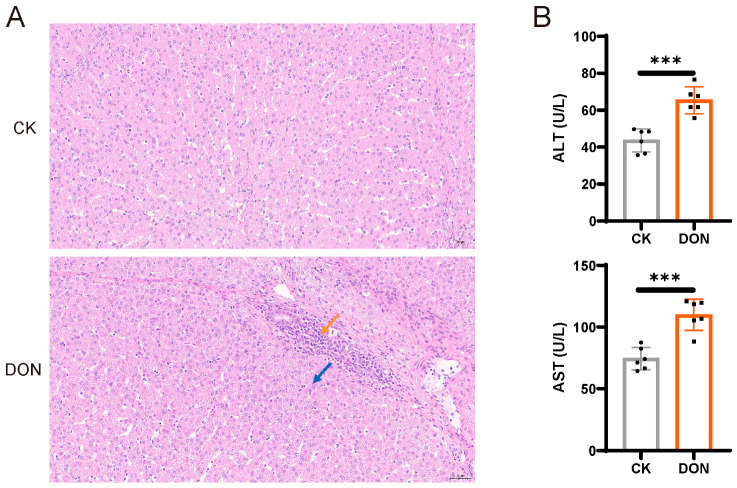
DON causes liver injury in piglets. (**A**). Representative histology of pig livers’ H&E staining. The blue arrows indicate cellular edema, and the orange arrows indicate lymphocyte infiltration. Scale bars: 100 µm. (**B**). ELISA measurement of ALT and AST in pig livers (n = 6). *** indicated *p* < 0.01.

To explore the effect of DON on liver injury in piglets, we established a model of piglets exposed to DON. Compared to the control group livers, Hematoxylin and Eosin (H&E) staining results showed mild edema in many hepatocytes and visible small focal infiltrations of lymphocytes, indicating an inflammatory response in the liver induced by DON treatment (Figure 1A). Simultaneously, we found significant changes in serum Alanine Aminotransferase (ALT) and Aspartate aminotransferase (AST) levels in the DON-treated piglets (Figure 1B), suggesting that DON caused liver injury in the piglets.

### 3.2. Protein Identification Using LC-MS/MS

**Figure 2:**
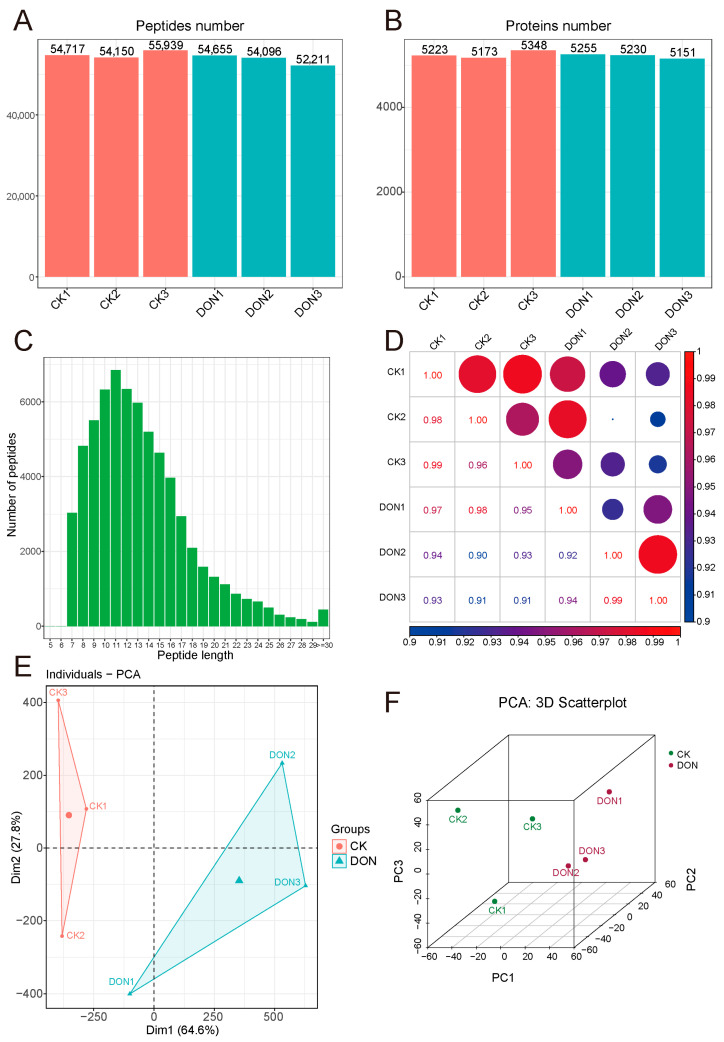
Proteomics analysis of porcine liver after DON treatment. (**A**,**B**) represent number of peptides and proteins detected in the two groups. (**C**). The peptide length profile shows the distribution of all identified peptide lengths. The abscissa represents the peptide length, and the ordinate represents the number of peptides detected. (**D**). Correlation analysis among different samples and groups. The size and clour represent the value of correlation. (**E**,**F**). Score plot of principal component analysis (PCA) of proteomics data from control and DON-treated livers in 2D and 3D.

We conducted quantitative proteomic sequencing analysis on the livers of healthy and DON exposed piglets. In total, 5851 proteins belonging to 65,745 peptides were identified by proteomics across all samples, and no fewer than 50,000 peptides and 5000 proteins were detected in each sample (Figure 2A–C), indicating a high abundance of proteins in the samples. Analysis of protein detection within each group showed that the three samples in the DON group shared 4767 common proteins, while the three CK (Control) samples shared 4811 common proteins (Figure S1A–C). Analysis of sample repeatability showed high intra-group correlation and lower inter-group correlation among samples (Figure 2D). Principal Component Analysis (PCA) results showed good separation between the two groups of samples in both two-dimensional and three-dimensional levels (Figure 2E,F), indicating good intra-group reproducibility and certain differences between the groups.

### 3.3. DON-Exposed Interfered Protein Expression in Liver

**Figure 3:**
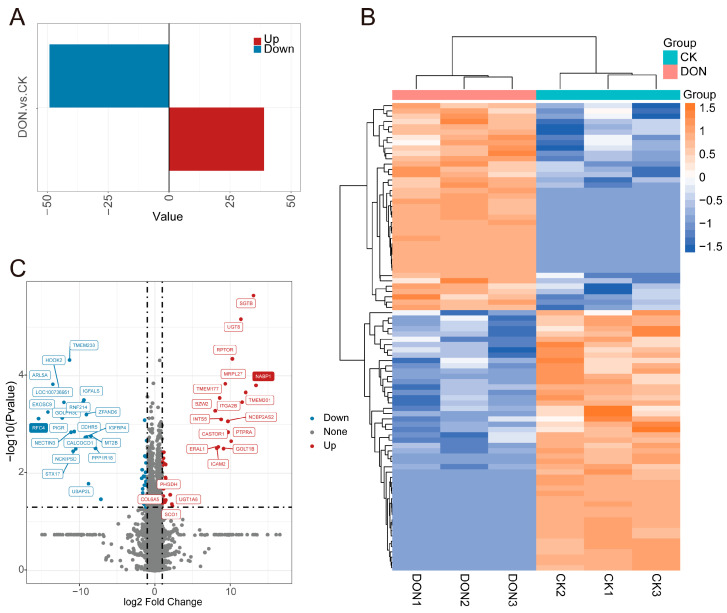
Differential expression proteins in livers after DON treatment. (**A**). Bar graph showing the number of upregulated and downregulated differentially expressed proteins. Red represents upregulated proteins, and blue represents downregulated proteins. (**B**). Heatmap of differentially expressed proteins. (**C**). Volcano plot showing differentially expressed proteins. Red represents upregulated proteins, blue represents downregulated proteins, and gray represents no significant difference.

**Figure 4:**
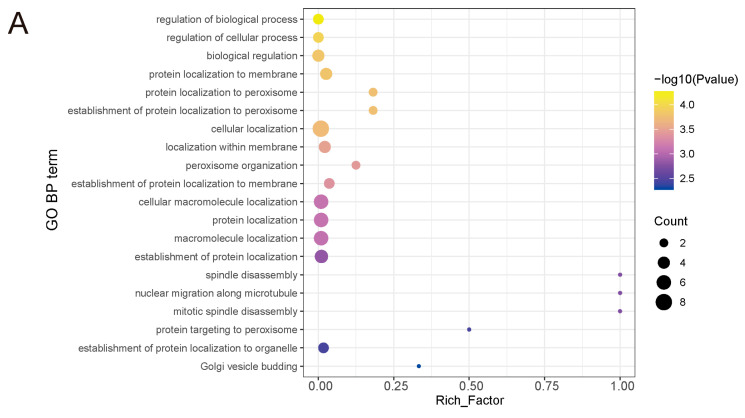

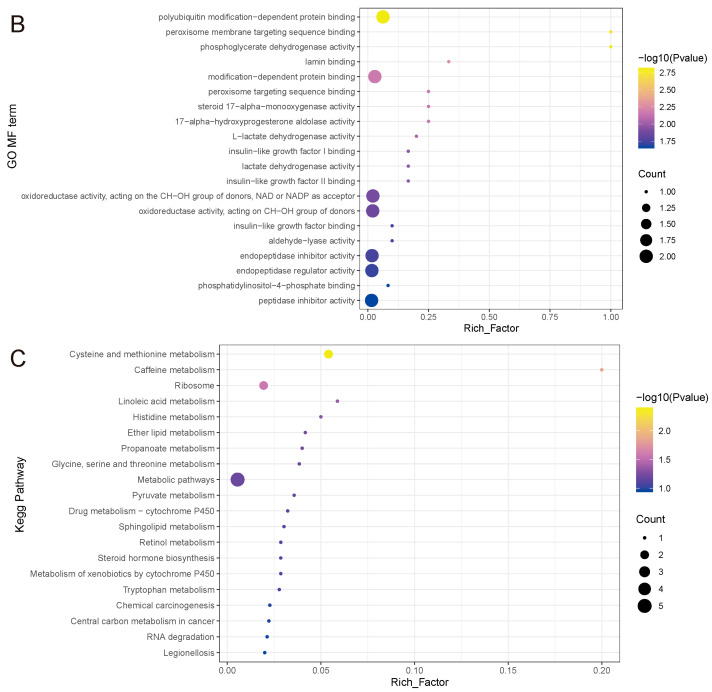
Functional enrichment of DEPs. (**A**–**C**). GO biological process, molecular function and KEGG enrichment analysis of differentially expressed proteins in control (CK) and DON-treated livers. The size of the dots represents the number of proteins, and the color represents the significance of the pathway. The x-axis indicates the enrichment factor, and the y-axis represents different functional terms.

We performed differential analysis on the 5851 detected proteins using a threshold of a 2-fold change and a *p*-value less than 0.05. A total of 88 proteins showed significant differences in expression levels after DON treatment (Figure 3A,B). Compared to the control group, 39 proteins were significantly upregulated and 49 were significantly downregulated. Among them, NABP1 and RFC4 were the most upregulated and downregulated proteins, with Log2FC values of 13.416 and −15.414, respectively (Figure 3C). The complete list is provided in Supplementary Table S1. SGTB and TMEM238 were the most statistically significant upregulated and downregulated proteins. Functional enrichment analysis was performed on all DEPs to understand their functions. GO enrichment analysis showed that Biological Process (BP) related functions were mainly enriched in the regulation of biological and cellular processes, protein localization, and peroxisome-related terms (Figure 4A). Molecular Function (MF) related functions mainly showed differences in polyubiquitin protein binding and various metabolic enzyme activities (Figure 4B). KEGG pathway analysis indicated that DEPs were mainly enriched in cysteine metabolism, caffeine metabolism, ribosome, and several metabolic pathways (Figure 4C). Although several metabolic pathways showed higher richness factors, we focused on the most statistically significant terms (lowest adjusted *p*-values) that directly aligned with the DNA/RNA regulatory hypothesis. In conclusion, we have demonstrated that DON exposure can induce liver damage by influencing protein expression.

**Figure 5:**
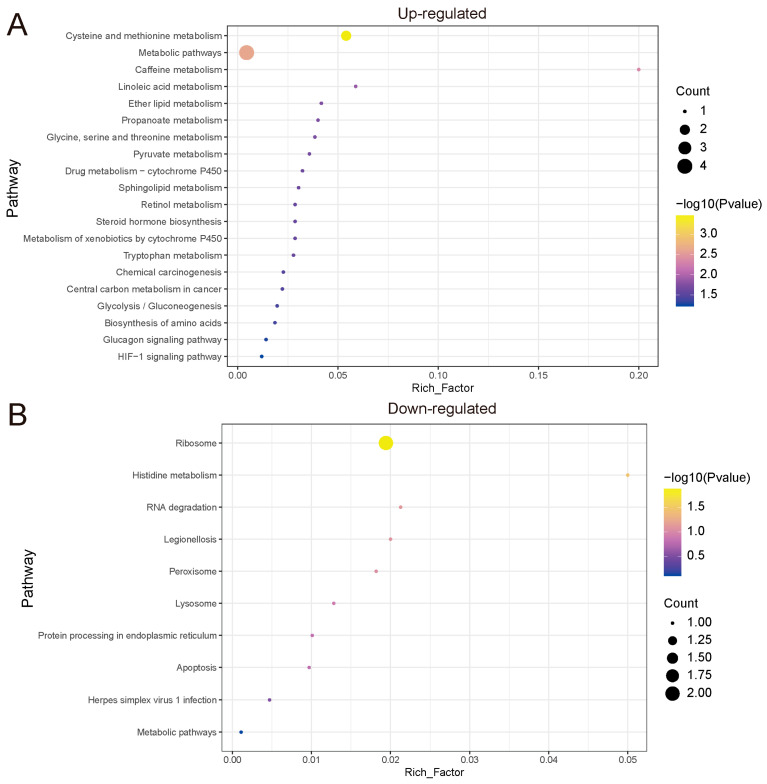
Functional enrichment of up- and down-regulated DEPs. (**A**,**B**) represent KEGG enrichment of up- and down-regulated DEPs. The size of the dots represents the number of proteins, and the color represents the significance of the pathway. The x-axis indicates the enrichment factor, and the y-axis represents different functional terms.

To further investigate the functions of the DEPs, separate in-depth functional enrichment analyses were performed on the upregulated and downregulated DEPs. The results showed that upregulated and downregulated proteins were involved in different functions and pathways. KEGG enrichment results showed that upregulated proteins were mainly involved in cysteine metabolism, caffeine metabolism, and related metabolic pathways, while downregulated proteins were primarily involved in ribosome and RNA degradation-related pathways (Figure 5A,B). GO enrichment analysis showed that in BP terms, upregulated proteins were mainly involved in nuclear migration, L-serine biosynthesis, and nuclear localization functions, while downregulated proteins were mainly involved in protein localization and peroxisome-related functions (Figure S2A,B).

### 3.4. Protein–Protein Interaction (PPI) Analysis

**Figure 6:**
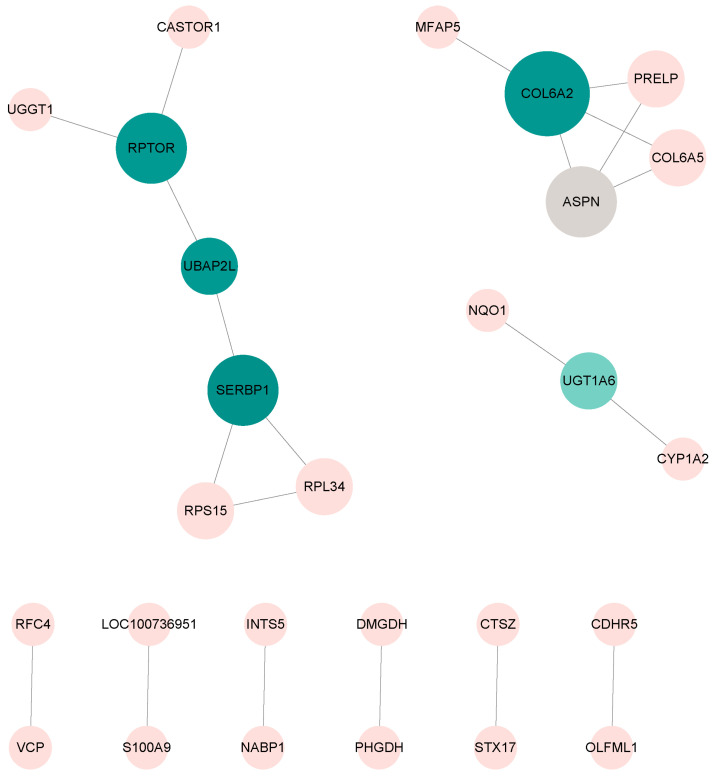
Protein–protein interactions between DEPs. Lines indicate the pairwise interaction relationships between proteins. The color of the circles represents the connectivity of the proteins, with blue indicating higher connectivity. The size of the circles represents the degree. Relationships between protein UGGT1, CASTOR1, RPTOR, UBAP2L, SERBP1, RPS15, RPL34, between MFAP5, COL6A2, PRELP, COL6A5, ASPN between, and NQO1 UGT1A6, between this CYP1A2. The proteins with pairwise interaction relationships are RFC4-VCP, LOC100736951-S100A9, INTS5-NABP1, DMGDH-PHGDH, CTSZ-STX17, and CDHR5-PLFML1, respectively.

A PPI network interaction map was constructed using the STRING database (STRING 12.5) and Cytoscape software (Cytoscape 3.10.4). The results showed that the DEPs were divided into different clusters of interacting proteins (Figure 6). Some proteins had certain interaction relationships, such as RPS15, RPL34, SERBP1, UBAP2L, RPTOR, UGGT1, and CASTOR1. Some proteins also had pairwise interactions, such as PHGDH and DMGDH.

### 3.5. Western Blot (WB) Validation

**Figure 7:**
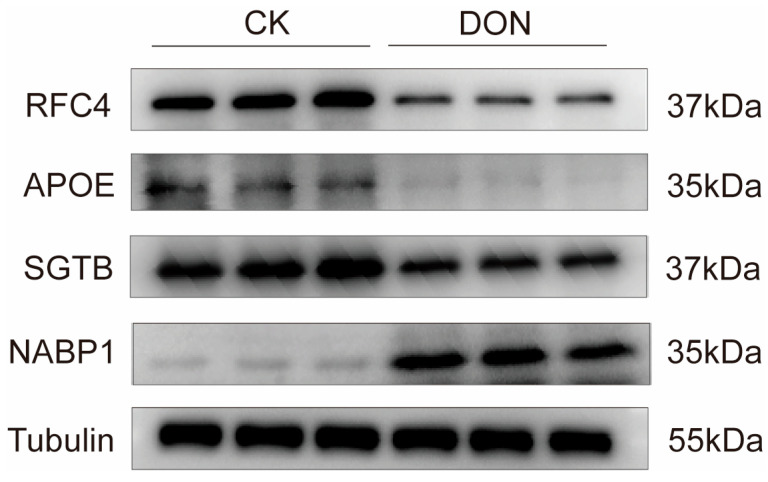
WB validate of proteomcis. Protein expression levels of APOE, RFC4, SGTB, NABP1 in porcine livers determined by WB, with Tubulin as the loading control (n = 3). The original WB image for this figure can be found in Figure S3.

To verify the expression of the key proteins discovered by proteomics sequencing in the liver under DON exposure, we selected the key proteins from the sequencing results for experimental verification by Western Blot (WB) experiments to validate the accuracy of the proteomic results. The results showed that after DON treatment, the expression levels of RFC4,SGTB and APOE proteins in liver samples were significantly decreased, while the expression level of NABP1 protein was significantly increased (Figure 7), indicating the accuracy of the proteomic results.

## 4. Discussion

Mycotoxins are significant factors endangering livestock production [27]. DON, as one of the most common mycotoxins in mainland China, has enormous negative impacts on the growth and development of piglets [28]. In addition to its damaging effects through the gastrointestinal tract, the liver is also injured by DON during metabolic processes, leading to liver damage through the induction of cell death pathways such as autophagy, ferroptosis, and pyroptosis [17,18,19]. To investigate how DON affects the pig liver proteome, we constructed the liver protein profiles before and after DON treatment. First, we found that DON exposure caused morphological damage to the liver tissue of piglets, indicating the successful establishment of a DON exposure liver injury model. Subsequently, our proteomic analysis identified 5851 proteins in total, with 88 being differentially expressed. Among the downregulated proteins, the one with the largest difference was Replication Factor C Subunit 4 (RFC4), a core component of the Replication Factor C (RFC) complex, primarily involved in DNA damage repair and is an important protein for maintaining genomic stability in hepatocytes [29]. This protein has been found to be negatively correlated with tumor prognosis; patients with poor prognosis had significantly higher RFC4 expression levels than those with good prognosis [30,31]. Arai et al. found that RFC4 knockdown inhibited hepatocyte proliferation [32]. This series of evidence aligns with our result where RFC4 showed the most significant response to DON. Recently, Li et al. proposed a “two-hit” phenomenon for mycotoxins, where they simultaneously disrupt cell function and inhibit cellular antioxidant repair, jointly damaging health. The significant decrease in RFC4 in our results provides a new direction for this theory, suggesting that the downregulation of RFC4 suggests that DON exposure may impair DNA repair capacity, preventing their self-recovery [27].

Besides RFC4, the second most downregulated protein was Exosome Component 9 (EXOSC9), a core component of the RNA exosome complex, widely involved in the processing and degradation of various RNAs within cells [33]. Mutations in EXOSC9 can cause a Pontocerebellar Hypoplasia (PCH)-like disease with cerebellar atrophy and spinal motor neuron disease. This protein is also positively correlated with tumor cell proliferation; Khan et al. found that FRG1 can activate EXOSC9 expression in this process [34].

Notably, DON exposure also increased the expression of some proteins. The most significantly upregulated protein was Nucleic Acid Binding Protein 1 (NABP1), also commonly known as hSSB1 (Human Single-Stranded DNA Binding Protein 1), an important single-stranded DNA-binding protein in humans. Its core function is to recognize and tightly bind to exposed single-stranded DNA regions in various DNA metabolic processes (such as replication, recombination, repair) [35]. This protein is also related to hepatocyte proliferation and can regulate the cell cycle and apoptosis in HK2 cells during acute kidney injury [36]. This might be a self-protection mechanism of hepatocytes against DON toxin, attempting to maintain cell activity by rapidly increasing expression. NABP1 can also undergo RNA methylation modification mediated by ZC3H13, an epigenetic modification that can stabilize its mRNA [37]. In this study, increased NABP1 expression could represent a compensatory cellular response to DNA stress. Carbon metabolism is an important metabolic pathway in the liver. LDHB is responsible for reducing pyruvate to lactate. A study on acetaminophen (APAP)-induced liver injury found that a decrease in SIRT1/PGC1-α/LDHB increased liver damage and led to increased lactate and protein lactylation levels [38]. This is a newly discovered metabolism-driven epigenetic modification that plays an important role in maintaining hepatocyte proliferation and DNA damage repair [39,40,41]. Upregulation of LDHB might indicate a potential link between DON-induced metabolic reprogramming and histone lactylation. The underlying mechanisms deserve further exploration in our subsequent work.

We performed separate functional enrichment analyses on the upregulated and downregulated proteins, which provided some hints worthy of in-depth study. The downregulated proteins were mainly enriched in the ribosome pathway, suggesting that DON injury might cause liver damage by inhibiting RNA translation. Translatomics, a popular technique in recent years that accurately reflects translation levels, can be used for correlation analysis with proteomics to determine the consistency between translation and protein levels [42]. Subsequently, we can use this technology to determine the impact of DON on the liver’s translational state for a more comprehensive understanding of DON’s damage mechanism. The upregulated proteins were most significantly enriched in the cysteine metabolism pathway. Cysteine participates in the synthesis of glutathione, the body’s primary antioxidant [43]. Since DON primarily causes damage through oxidative stress, this phenomenon might represent the body’s spontaneous therapeutic response. Although ribosome-related proteins were globally downregulated, indicating translational suppression, certain stress-response proteins such as NABP1 and LDHB were upregulated, likely through selective translation mechanisms that prioritize cellular repair and survival during DON-induced stress. However, this study still has its limitations. For instance, we only conducted protein sequencing analysis and did not delve deeply into the mechanism of how the identified proteins induce liver damage caused by DON exposure. This requires us to explore it in detail in the subsequent research.

## 5. Conclusions

This study provides exploratory proteomic evidence revealing novel molecular mechanisms underlying DON-induced hepatotoxicity in piglets. The core finding is that DON induces toxicity by significantly affecting pathways related to DNA damage repair (e.g., RFC4 downregulation), RNA metabolism (e.g., EXOSC9 downregulation), and ribosome function, disrupting hepatocyte genomic stability and internal homeostasis. Although the body may attempt compensation by upregulating proteins like NABP1, it is insufficient to reverse the damage. These findings not only deepen the understanding of DON hepatotoxicity mechanisms, particularly by clarifying key changes at the protein level, but also provide an important theoretical basis and molecular targets for subsequent toxicological research, biomarker discovery, and feed safety risk assessment.

## Data Availability

Data are available at National Genomics Data Center with Bioproject PRJCA049010.

## Supplementary Materials

The following supporting information can be downloaded at: https://www.mdpi.com/article/10.3390/biology14121721/s1, Figure S1: Protein identified with proteomics; Figure S2: GO BP enrichment of up- and down-regulated DEPs; Figure S3. WB original images of Figure 7; Table S1: Differential expression proteins after DON treatment.

## Abbreviations

The following abbreviations are used in this manuscript: 3-ADON: 3-acetyl-deoxynivalenol; 15-ADON: 15-acetyl-deoxynivalenol; AGC: Automatic Gain Control; ALT: Alanine Aminotransferase; APOE: Apolipoprotein E; BCA: Bicinchoninic Acid; BP: Biological Process (Gene Ontology); CK: Control Group; CYP450: Cytochrome P450; DDA: Data-Dependent Acquisition; DEPs: Differentially Expressed Proteins; DIA: Data-Independent Acquisition; DON: Deoxynivalenol; DTT: Dithiothreitol; EP tube: Microcentrifuge tube; EXOSC9: Exosome Component 9; FC: Fold Change; GB: Guobiao (Chinese National Standard); GO: Gene Ontology; H&E: Hematoxylin and Eosin; IAM: Iodoacetamide; KEGG: Kyoto Encyclopedia of Genes and Genomes; LC-MS/MS: Liquid Chromatography-Tandem Mass Spectrometry; LDHB: Lactate Dehydrogenase B; MAPK: Mitogen-Activated Protein Kinase; MF: Molecular Function (Gene Ontology); MS: Mass Spectrometry; m/z: Mass-to-charge ratio; NABP1: Nucleic Acid Binding Protein 1; NF-κB: Nuclear Factor Kappa-B; NH4HCO3: Ammonium Bicarbonate; PCA: Principal Component Analysis; PMSF: Phenylmethanesulfonyl fluoride; PPI: Protein–Protein Interaction; PRM: Parallel Reaction Monitoring; RFC4: Replication Factor C Subunit 4; rpm: Revolutions per minute; SP3: Single-pot solid-phase-enhanced sample preparation; TMT: Tandem Mass Tag; WB: Western Blot.
