# Supplementary material for: Proteomic Analysis of Liver Injury Induced by Deoxynivalenol in Piglets
Source: Biology (Basel). 2025 Dec 1;14(12):1721. doi: 10.3390/biology14121721 (PMC12730578; doi:10.3390/biology14121721)
Supplement: Supplementary file 1 [file biology-14-01721-s001.zip › Sup Figs.pdf]

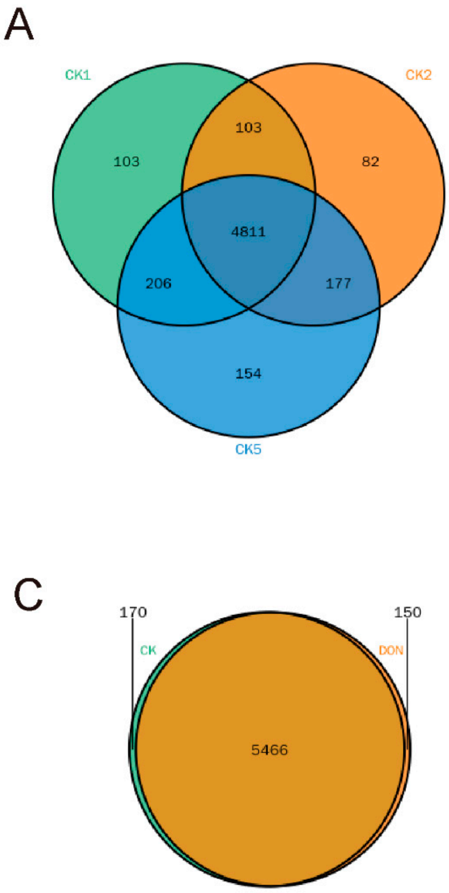

Supplementary Figure S1. Protein identified with proteomics. A, B. Numbers of proteins in each sample. C. Number of proteins in each groups.

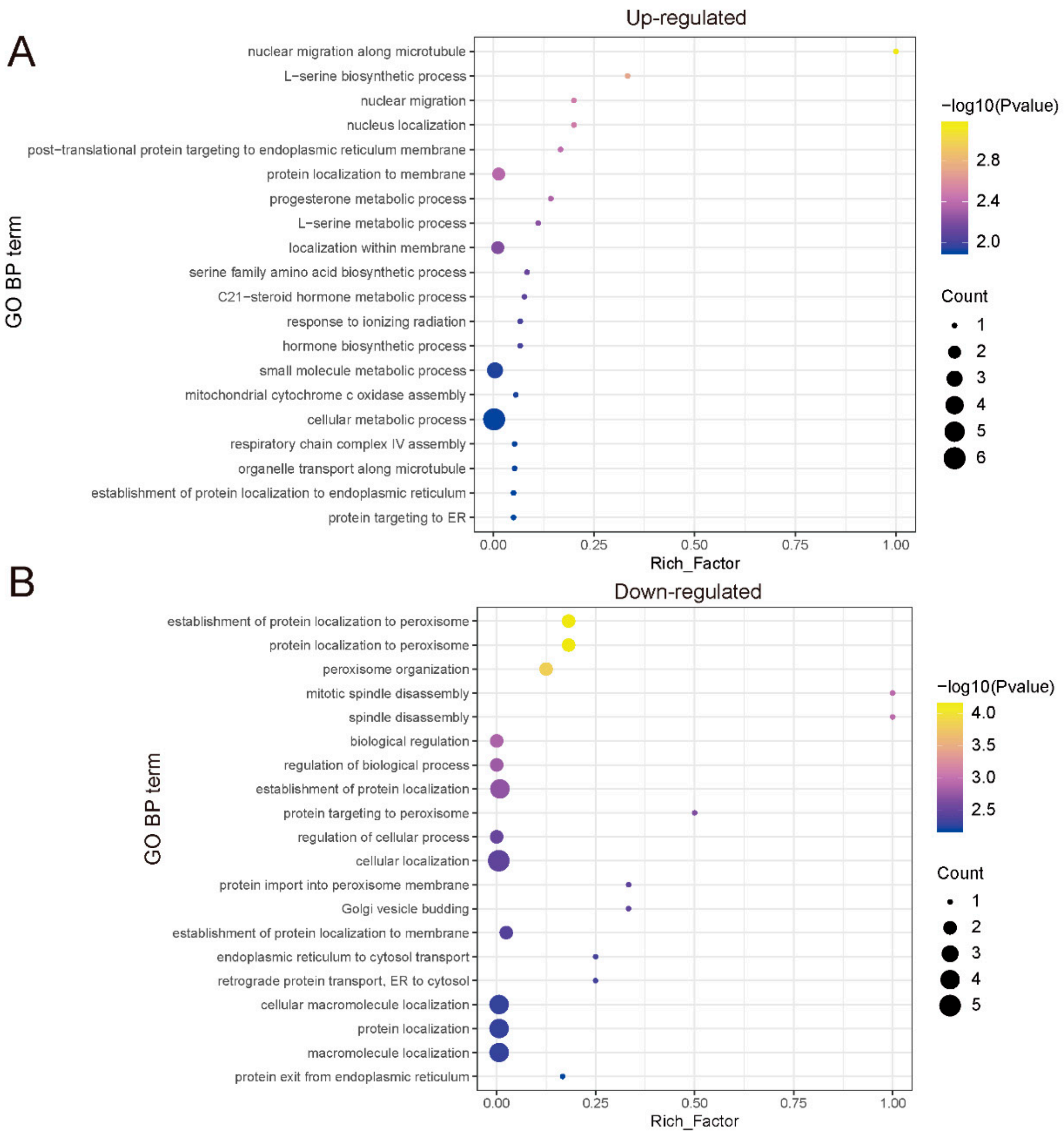

Supplementary Figure S2. GO enrichment of up- and down-regulated proteins.

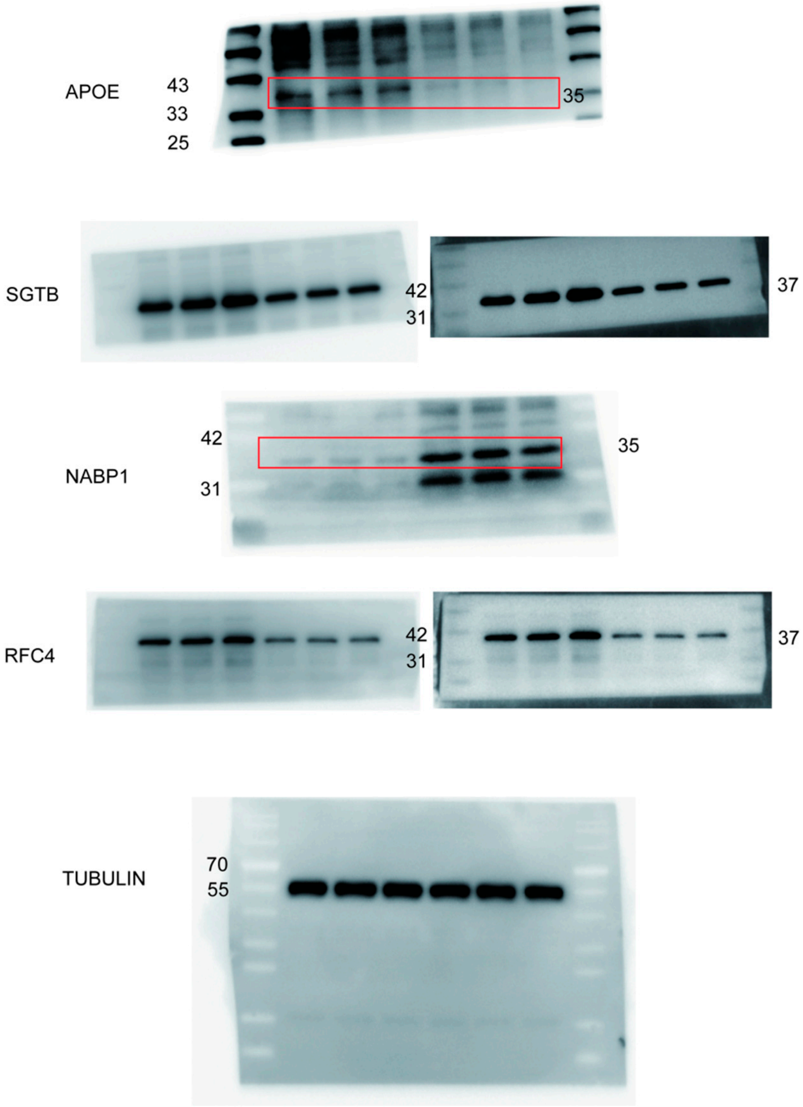

Supplementary Figure S3. WB original images of Figure 7.
