# Supplementary material for: Proteomic Analysis of Liver Injury Induced by Deoxynivalenol in Piglets
Source: Biology (Basel). 2025 Dec 1;14(12):1721. doi: 10.3390/biology14121721 (PMC12730578; doi:10.3390/biology14121721)
Supplement: Supplementary file 1 [file biology-14-01721-s001.zip › Table S1.pdf]

Table S1. Differential expression proteins after DON treatment.

Protein ID	Log_2FC	P-value	sign	Gene Name
A0A4X1SHF0	13.41649831	0.000157316	Up	NABP1
A0A286ZNJ9	13.08149241	2.24601E-06	Up	SGTB
A0A286ZM85	12.05966552	0.000219998	Up	TMEM201
A0A5G2QIS2	11.57889801	0.000347709	Up	ITGA2B
I3LFS4	11.41660698	6.897E-06	Up	UGT8
I3L942	10.28342711	4.49299E-05	Up	RPTOR
A0A287AV68	10.12089194	0.002204491	Up	PTPRA
A0A287AIJ6	9.70644789	0.001442138	Up	CASTOR1
A0A8W4FBZ7	9.687731821	0.000861828	Up	NCBP2AS2
A0A8D0PPM8	9.342589281	0.000146337	Up	MRPL27
A0A5G2QSY4	9.116286665	0.003142669	Up	GOLT1B
I3LMH3	8.796999833	0.000785864	Up	INTS5
F1RXW5	8.595751063	0.000286063	Up	TMEM177
F1RNA7	8.423231837	0.002883452	Up	ERAL1
A0A287B7Y4	8.216031996	0.003070757	Up	ICAM2
A0A286ZT34	8.014739278	0.000524188	Up	BZW2
A0A287B177	2.335064343	0.047321719	Up	UGT1A6
A0A287ACH8	2.267796597	0.043188253	Up	SCO1
A5GFY8	2.043122962	0.027793775	Up	PHGDH
A0A287BK35	1.469000871	0.035822455	Up	COL6A5
A0A286ZYK5	1.456134372	0.012592103	Up	FCSK
A0A5G2QIX0	1.450751326	0.006640467	Up	MRPS2
A0A5G2Q9G3	1.413074652	0.012327064	Up	ART4
A0A5G2R2Y0	1.355557595	0.016884244	Up	MFAP5
A0A287A1T6	1.318706954	0.039641119	Up	COL6A5
F1SJ26	1.306490854	0.017192223	Up	CYP1A2
A0A8W4FGK0	1.296412517	0.036126783	Up	SUSD2
A0A5G2Q9I0	1.289742845	0.006405817	Up	UBE2L6
A0A4X1SHU0	1.199644255	0.003717537	Up	NQO1
A0A5G2QYG4	1.174363823	0.035981219	Up	PDGFRB
P00336	1.158890019	0.020780539	Up	LDHB
F1SUE4	1.147009206	0.004939936	Up	ASPN
F1S6B4	1.124950973	0.029487862	Up	PRELP
A0A8D1I2F1	1.084608754	0.003794221	Up	LGALS3
I3LQ84	1.068877075	0.041013388	Up	COL6A2
F1SBZ3	1.050134105	0.005912458	Up	HYCC1
A0A286ZLN5	1.043971099	0.00688008	Up	KYNU
A0A286ZY90	1.023235001	0.03639396	Up	OLFML1
F1RF50	1.002307814	0.035408123	Up	DMGDH
A0A287AE58	-1.006028778	0.016294805	Down	CD5L
A0A075B7I6	-1.081232462	0.002145944	Down	None
P80054	-1.12693831	0.014541553	Down	PR39

A0A287AUR6	-1.131441035	0.040127553	Down	GLOD5
A0A8D1AXG3	-1.13453436	0.017853656	Down	ATP5F1D
A5GFX7	-1.16400276	0.006159938	Down	CTSZ
F6Q2M9	-1.188897649	0.015049931	Down	FAM210B
A0A8W4FQM2	-1.201648897	0.004615248	Down	CALU
A0A4X1VMF9	-1.202016343	0.004451999	Down	TMEM258
F1RM45	-1.204035959	0.049768382	Down	APOE
A8WH75	-1.233920731	0.009629859	Down	CD302
Q29076	-1.265918156	0.030814393	Down	POU2F1
A0A4X1T0W5	-1.27115795	0.011838216	Down	TMA7
F1RNA5	-1.30081065	0.039355211	Down	DHRS13
K7GME6	-1.330844589	0.038241974	Down	S100A9
I3LRG2	-1.333820737	0.004450469	Down	PEX19
P62844	-1.350521815	0.000818744	Down	RPS15
A0A5G2RD08	-1.393445722	0.017695467	Down	UGGT1
F1S126	-1.399656789	0.031158196	Down	ETNPPL
A0A287AK40	-1.402687456	0.043805825	Down	None
P79378	-1.486765161	0.042026499	Down	MT1F
F1S827	-1.489634692	0.011597307	Down	SERBP1
P79263	-1.595669296	0.025585594	Down	ITIH4
A0A5G2R9F7	-1.602199565	0.02106229	Down	FKBP1A
A0A287AA45	-1.617493429	0.040322534	Down	HNMT
A0A287BRL8	-1.68006697	0.008515334	Down	MARCKS
Q29223	-1.735359492	0.012765357	Down	RPL34
A0A8W4FK22	-5.083288267	0.001231065	Down	PCNA
P03974	-7.146621021	0.034447389	Down	VCP
Q767M0	-7.865450163	0.003093357	Down	PPP1R18
P79380	-8.483880418	0.001734522	Down	MT2B
A0A286ZKU8	-8.79828717	0.01652021	Down	UBAP2L
P24854	-9.030399855	0.001814374	Down	IGFBP4
A0A287A149	-9.068653456	0.000627287	Down	ZFAND6
A0A287AYQ8	-9.206694446	0.001850364	Down	CALCOCO1
Q1KS52	-9.39888177	0.000314617	Down	IGFALS
A0A286ZMT3	-9.496335758	0.000350973	Down	RNF214
A0A287AQL8	-10.47466015	0.003165335	Down	STX17
A0A287AHX7	-10.6890766	0.00140106	Down	CDHR5
A0A480QVB2	-10.82507523	0.00355014	Down	NCKIPSD
A0A8D1FRE1	-10.98610874	0.000485487	Down	GOLPH3L
K7GMN4	-11.11401113	0.001445527	Down	NECTIN3
A0A287BCQ2	-11.30308615	4.74281E-05	Down	TMEM238
F1SNU4	-12.04741571	0.000350439	Down	LOC100736951
A0A287A0N2	-12.27977093	0.000736437	Down	PIGR
A0A287BM99	-12.65844018	0.00020088	Down	HOOK2
A0A287A3R6	-13.51627384	0.000149348	Down	ARL5A

F1S1T6	-14.17816518	0.000556908	Down	EXOSC9
A0A287AQT0	-15.41449664	0.000758544	Down	RFC4
